# Expression of lactylation-related genes and their correlation with diabetic foot ulcer occurrence and immune infiltration

**DOI:** 10.3389/fimmu.2026.1765123

**Published:** 2026-04-29

**Authors:** Xiaolong Hu, Junpeng Zhou, Meng Guo, Wei Peng, Chen Yang, Fang Wang, Wei Zhang, Jiaqi Liu

**Affiliations:** 1 Department of Burns and Cutaneous Surgery, Xijing Hospital, Air Force Medical University, Xi’an, China; 2 Department of Plastic, Burn & Medical Cosmetic Surgery, The First Affiliated Hospital of Xi’an Medical University, Xi’an, China; 3 Medical innovation research platform, Qidong-Fudan Innovative Institute of Medical Sciences, Fudan University, Shanghai, China

**Keywords:** biomarker, diabetic foot ulcers, immune infiltration, lactylation, machine learning

## Abstract

**Background:**

Diabetic foot ulcers (DFUs) represent a severe complication of diabetes, often leading to chronic non-healing wounds and high amputation risk. Lactylation, a recently recognized post-translational modification driven by lactate metabolism, has emerged as a key regulator of immune response and gene expression. However, its role in DFU pathogenesis remains largely unexplored. This study aims to systematically investigate lactylation-related genes and their association with immune dysregulation in DFUs.

**Methods:**

Transcriptomic data from three GEO datasets (GSE134431, GSE80178, GSE68183) were integrated and normalized to identify differentially expressed genes (DEGs). A lactylation-related gene set was compiled from published literature. Machine learning approaches, including LASSO regression and Random Forest, were applied to screen for core genes. Immune infiltration profiles were assessed using ssGSEA. Experimental validation was conducted in high-glucose-stimulated macrophages and human DFU tissues via qPCR, Western blot, immunohistochemistry, and immunofluorescence.

**Results:**

We integrated three transcriptomic datasets comprising 25 DFU and 14 normal tissues, identifying 1,234 differentially expressed genes (DEGs). Among these, 38 overlapped with lactylation-related genes, with 27 significantly downregulated in DFU. Machine learning algorithms identified three core lactylation-associated genes: CHD4, EEF1A1, and EEF1G, which exhibited significant downregulation in DFU and demonstrated high within cohort classification performance with AUC values of 0.860, 0.926, and 0.989, respectively. Immune infiltration analysis revealed these genes positively correlated with natural killer cells and negatively correlated with neutrophil infiltration. Experimental validation in high glucose-treated macrophages and human DFU tissues confirmed their reduced expression at both transcriptional and protein levels, particularly noting marked loss of EEF1A1 in epidermal layers and infiltrating CD68+ macrophages. Direct measurement of lysine lactylation (Kla) confirmed increased global lactylation under diabetic conditions.

**Conclusion:**

This study identifies CHD4, EEF1A1, and EEF1G as key lactylation-related genes involved in DFU progression, with significant classificational potential and close links to immune microenvironment dysregulation. These findings highlight lactylation as a promising regulatory mechanism in diabetic wound pathology and support further development of lactylation-targeted biomarkers and therapeutic strategies for DFU management, and require external validation and functional mechanistic studies.

## 1 Introduction

Diabetic Foot Ulcers (DFUs) represent one of the most challenging and prevalent complications in patients with diabetes (1). The primary contributing factors for DFUs include prolonged hyperglycemia, which leads to peripheral neuropathy and vascular disease, thereby creating an environment conducive to the development of non-healing ulcers on the feet of diabetic patients (2, 3). DFUs carry serious implications, as they commonly lead to severe infections, substantially reduce the quality of life, and in extreme cases necessitate lower limb amputations (4). Consequently, early diagnosis coupled with effective treatment of DFUs is critically important to prevent adverse outcomes and improve patient prognosis (5).

A growing body of research has underscored the importance of lactylation, a novel form of post-translational modification involving the attachment of lactate molecules to proteins (6). Lactate, the precursor for lactylation, is a byproduct of aerobic glycolysis, also known as the Warburg effect, a metabolic phenomenon frequently observed in cancer cells but also relevant in other pathological and physiological states (7). Recent studies have elucidated that lactate is not merely a metabolic byproduct; instead, it plays a critical role in epigenetic modifications. Specifically, lactylation involves the addition of a lactyl group to the ϵ-nitrogen of lysine residues on proteins, particularly histones, a modification known as lysine lactylation (Kla). This modification significantly influences chromatin structure and gene transcription regulation. Histone proteins, when modified by lactylation, participate in several vital physiological processes. Evidence suggests that Kla can influence immune cell homeostasis (8, 9). Immune regulation is paramount in chronic diseases like diabetes, where immune dysregulation further complicates the clinical scenario. Lactylation-modified histones have been shown to assist in the transition of macrophages from an inflammatory state to a reparative state, a transition crucial for effective wound healing, suggesting that lactylation can modulate immune responses that promote tissue repair (10). Furthermore, lactylation is implicated in stem cell differentiation, emphasizing its importance in tissue regeneration processes. Early embryonic development also leverages histone lactylation to ensure the proper expression of genes necessary for correct differentiation and development (11).

In pathological contexts, increased levels of histone lactylation have been linked to various diseases, suggesting its potential as a biomarker and therapeutic target. For instance, higher levels of Kla have been observed in conditions such as ulcerative colitis, pulmonary fibrosis, sepsis, systemic lupus erythematosus, and ocular tumors (12–15). These associations emphasize lactylation’s potential role in disease regulation and therapy. In ulcerative colitis, chronic inflammation of the colon involves gene expression changes driven by Kla, hinting at its role in inflammatory modulation. Similarly, in pulmonary fibrosis, histone lactylation induced by lactate in lung myofibroblasts promotes macrophage activity that fosters fibrosis, suggesting a broader role of lactylation in chronic non-healing wounds like DFUs (16). Although research into the specific mechanisms by which lactylation influences DFUs is still in its early stages, several key areas of potential impact are emerging.

Diabetic patients frequently exhibit metabolic abnormalities, including lactate accumulation. Lactylation modifications could alter critical metabolic pathways within cells, impacting wound healing processes. Lactate accumulation in the tissues of diabetic patients might lead to increased lactylation, potentially promoting or hindering cellular repair mechanisms depending on the context (17). Lactate has been identified as a pivotal regulator of immune responses. Lactylation modifications could significantly modulate the inflammatory milieu at the ulcer site by influencing the expression and function of proteins involved in inflammatory responses (18, 19). Chronic inflammation is a hallmark of non-healing diabetic ulcers. Thus, understanding how lactylation modulates inflammation could provide insights into interrupting the cycle of chronic inflammation and impaired healing. Lactylation might interfere with various cell signaling mechanisms crucial for wound healing. For instance, angiogenesis—the formation of new blood vessels—is essential for supplying nutrients and oxygen to healing tissue (20). If lactylation affects key regulatory proteins involved in these pathways, it could either enhance or delay wound repair. By modifying histones and other proteins involved in gene regulation, lactylation can influence the expression of genes crucial for wound healing. These modifications might alter cellular behaviors such as proliferation, migration, and differentiation, all vital for tissue repair and regeneration. Given the often-impaired cellular functions in diabetic conditions, understanding how lactylation influences these processes could reveal new therapeutic targets.

Despite the research on the relationship between DFUs and lactylation modifications being in early stages, further exploration of this connection holds significant potential. Analyzing the expression profiles of lactylation-related genes in DFUs versus normal tissues could identify novel biomarkers or therapeutic targets. This approach could enable the development of targeted interventions aimed at modulating lactylation to promote healing.

The present study aims to explore the expression levels of lactylation-related genes in DFUs and normal tissues using transcriptomic data from the GEO database. By identifying differentially expressed lactylation-related genes, we hope to uncover key regulatory factors involved in DFU pathogenesis. Furthermore, we will investigate the relationship between these genes and immune cell infiltration, as the immune microenvironment plays a crucial role in wound healing. Understanding these interactions could offer a comprehensive view of the molecular mechanisms underlying DFUs and illuminate potential therapeutic targets.

For instance, immune cells such as macrophages play a decisive role in wound healing and tissue repair (21). The pro-inflammatory and anti-inflammatory phases of macrophages are critical for initiating and resolving inflammation and promoting tissue remodeling. Dysregulation of this balance can result in chronic wounds characteristic of DFUs. In this context, understanding how lactylation impacts macrophage polarization and function becomes essential. Emerging evidence suggests that lactylation regulates these processes, making it a promising area of research for novel therapeutic targets aimed at resolving chronic inflammation and promoting healing.

Additionally, the interplay between lactylation and other post-translational modifications (PTMs) such as acetylation, phosphorylation, and ubiquitination could provide further insights into the complex regulation of gene expression during wound healing. Each of these PTMs plays distinct yet interconnected roles in regulating protein function, stability, and interactions. By mapping out the crosstalk between these modifications in the context of DFUs, it may be possible to identify key regulatory nodes that could be targeted for therapeutic intervention.

Furthermore, the modulation of lactylation through pharmacological agents opens new avenues for therapeutic development. Developing small molecule inhibitors or activators of enzymes involved in lactylation could fine-tune this modification, restoring normal gene expression profiles in DFU tissues. These strategies could complement existing therapeutic approaches, providing a multi-targeted approach to treating these chronic wounds.

## 2 Materials and methods

### 2.1 Sample and transcriptomic data

We obtained transcriptomic data of diabetic foot ulcer (DFU) samples and their control samples from three different GEO datasets. The datasets included GSE134431 (GPL18573 platform, Disease: 13, Control: 8), GSE80178 (GPL16686 platform, Disease: 9, Control: 3), and GSE68183 (GPL16686 platform, Disease: 3, Control: 3). These datasets collectively comprise the sample types and candidate genes necessary for this study. In total, 25 DFU cases and 14 control cases were included in the subsequent analysis.

### 2.2 Human specimens

Skin wound specimens were obtained from the Department of Burns and Cutaneous Surgery at Xijing Hospital, Air Force Medical University. The cohort comprised three non−diabetic control subjects and three diabetic patients presenting with diabetic foot ulcers (DFU). From each subject, a 3–5 mm tissue section was excised from the wound margin for subsequent immunohistochemical and immunofluorescence analysis. The study protocol received approval from the Ethics Committee of Xijing Hospital, Air Force Medical University, and written informed consent was provided by all enrolled participants.

### 2.3 Lactylation-related genes and differential analysis

From various research articles, we extracted a total of 336 lactylation-related genes (**Supplementary Table S1**) (22–24). To tackle batch effects and integrate the different GEO datasets, we employed the R packages “limma” and “sva” for principal component analysis (PCA). This step helps in identifying and removing batch effects. The R package preprocessCore was used for data normalization. We performed differential expression analysis using the R package limma. For visual representation and analysis of individual gene expression differences across diverse samples, we utilized the R package ggplot2.

### 2.4 Gene function enrichment and annotation

We carried out GO annotation and KEGG enrichment analysis on differentially expressed genes using the R package “clusterProfiler.” (25) GO annotation was categorized and displayed in three domains: Biological Processes (BP), Cellular Components (CC), and Molecular Function (MF).

### 2.5 Target gene identification and interaction analysis

To identify potential target genes, we intersected the differentially expressed genes from regular transcriptomic analysis with the lactylation gene set. The intersected genes were subsequently annotated. For predicting the protein-protein interaction networks (PPI) of the identified genes, we used the STRING database (https://string-db.org/). Key differentially expressed genes were filtered through LASSO regression, facilitated by the R package glmnet. Additionally, core genes were determined using the random forest algorithm in the R package randomForest, which ranks genes based on importance. A detailed flow diagram of the entire bioinformatic workflow is provided in **Supplementary Figure S1A**. Software versions (R 4.3.1, limma 3.58, sva 3.50, glmnet 4.1-7, randomForest 4.7-1.1, etc.) and random seeds (set.seed (123) for all random processes).

### 2.6 Immune infiltration assessment

Immune cell infiltration levels were evaluated using the ssGSEA function in the R package GSVA. The R package clusterProfiler was employed to perform GSEA analysis on the correlation results.

### 2.7 Gene and gene-compound interaction analysis

The PPI interactions of three core genes were analyzed using the GENEMANIA database (http://genemania.org/). Upstream miRNAs and transcription factors of the genes were predicted using the regnetwork database. The networks were then visualized using Cytoscape software. To elucidate gene-compound interactions, we utilized the NetworkAnalyst database (https://www.networkanalyst.ca/).

### 2.8 Cell culture and high-glucose treatment

Bone marrow-derived macrophages (BMDMs) were isolated from the femurs and tibias of C57BL/6J mice (6–8 weeks old) and differentiated in DMEM complete medium supplemented with 10% fetal bovine serum (FBS, Gibco) and 20 ng/mL macrophage colony-stimulating factor (M-CSF, Sino Biological) for 7 days. Mature BMDMs were then randomized into two groups: a normal glucose control group (5.5 mM D-glucose) and a high glucose treatment group (25 mM D-glucose), and cultured for an additional 48 hours to simulate the diabetic stress microenvironment. Six male C57BL/6J mice (6–8 weeks old) were used for BMDM isolation. All animal procedures were approved by the Institutional Animal Care and Use Committee (IACUC) of Air Force Medical University (Approval No: KY20253558-1).

### 2.9 RNA extraction and quantitative real-time PCR

Total RNA was extracted from treated BMDMs using TRIzol reagent (Invitrogen) according to the manufacturer’s instructions. cDNA was synthesized from 1 μg of total RNA using the PrimeScript RT Master Mix (Takara). Quantitative PCR was performed using the SYBR Green Premix Pro Taq HS qPCR Kit (Accurate Biology) on a QuantStudio 5 Real-Time PCR System (Applied Biosystems). Gene expression levels were normalized to Actin and calculated using the 2^(-ΔCt) method. The primer sequences used are listed in **Supplementary Table S1**.

### 2.10 Western blot analysis

Total protein was extracted from BMDMs using RIPA lysis buffer containing PMSF. Protein concentration was determined using the BCA Protein Assay Kit. Equal amounts of protein were separated by SDS-PAGE and transferred onto PVDF membranes. After blocking with 5% non-fat milk, the membranes were incubated overnight at 4 °C with primary antibodies against EEF1A1 (1:1000, Abmart, PK02092S) and Actin (1:5000, biosharp, BL005B). For detection of global lysine lactylation (Kla), protein lysates from human acute wound and DFU tissues, as well as from mouse bone marrow−derived macrophages (BMDMs) were separated by SDS−PAGE and transferred onto PVDF membranes. After blocking with 5% non−fat milk, the membranes were incubated overnight at 4 °C with a pan−anti−lysine lactylation antibody (Thermo Fisher Scientific, PA5−116901, dilution 1:1,000). Subsequently, membranes were incubated with corresponding horseradish peroxidase-conjugated secondary antibodies at room temperature for 1 hour. Protein bands were visualized using an ECL chemiluminescence kit (beyotime) and imaged with a Tanon imaging system. Band intensities were quantified using ImageJ software.

### 2.11 Immunohistochemistry and immunofluorescence staining

For immunohistochemistry (IHC) analysis, paraffin-embedded tissue sections were deparaffinized, rehydrated, and subjected to antigen retrieval. Endogenous peroxidase activity was quenched with 3% hydrogen peroxide. After blocking with 3% BSA, the sections were incubated overnight at 4 °C with a primary antibody against EEF1A1 (1:500, Abmart, PK02092S). Subsequently, the sections were incubated with an HRP-conjugated secondary antibody, followed by development with DAB substrate and counterstaining with hematoxylin. Stained sections were visualized under a light microscope.

For immunofluorescence (IF) double staining, paraffin-embedded tissue sections were similarly processed through deparaffinization, rehydration, and antigen retrieval. After blocking with 3% BSA, the sections were incubated overnight at 4 °C with the following primary antibodies: rabbit anti-CD68 (1:200, Abcam, ab201340), mouse anti-EEF1A1 (1:500, Abmart, PK02092S), mouse anti-CD56 (1:200, Thermo Fisher Scientific, 740093M) and rabbit anti- Myeloperoxidase (MPO) (1:500, Abcam, ab208670). The sections were then incubated with Alexa Fluor 488-conjugated goat anti-mouse IgG and Alexa Fluor 594-conjugated goat anti-rabbit IgG (both from Invitrogen, 1:500) at room temperature for 1 hour in the dark. Nuclei were counterstained with DAPI. Images were captured using a Zeiss LSM 880 confocal microscope.

### 2.12 Statistical analysis

In comparing gene expression between disease (DFU) and control samples, differentially expressed genes were selected based on the criteria: adj.P.Val < 0.05 and |logFC| > 0.5. Spearman’s rank correlation analysis was performed among the core genes using normalized expression values from the combined GEO dataset. All statistical analyses were performed using R (version 4.3.1). Significant differences in immune cell infiltration between disease and control groups were identified based on the following thresholds: ns, p > 0.05; *p < 0.05, **p < 0.01, ***p < 0.001.

## 3 Results

### 3.1 GEO data normalization and differentially expressed genes analysis

**Figure 1:**
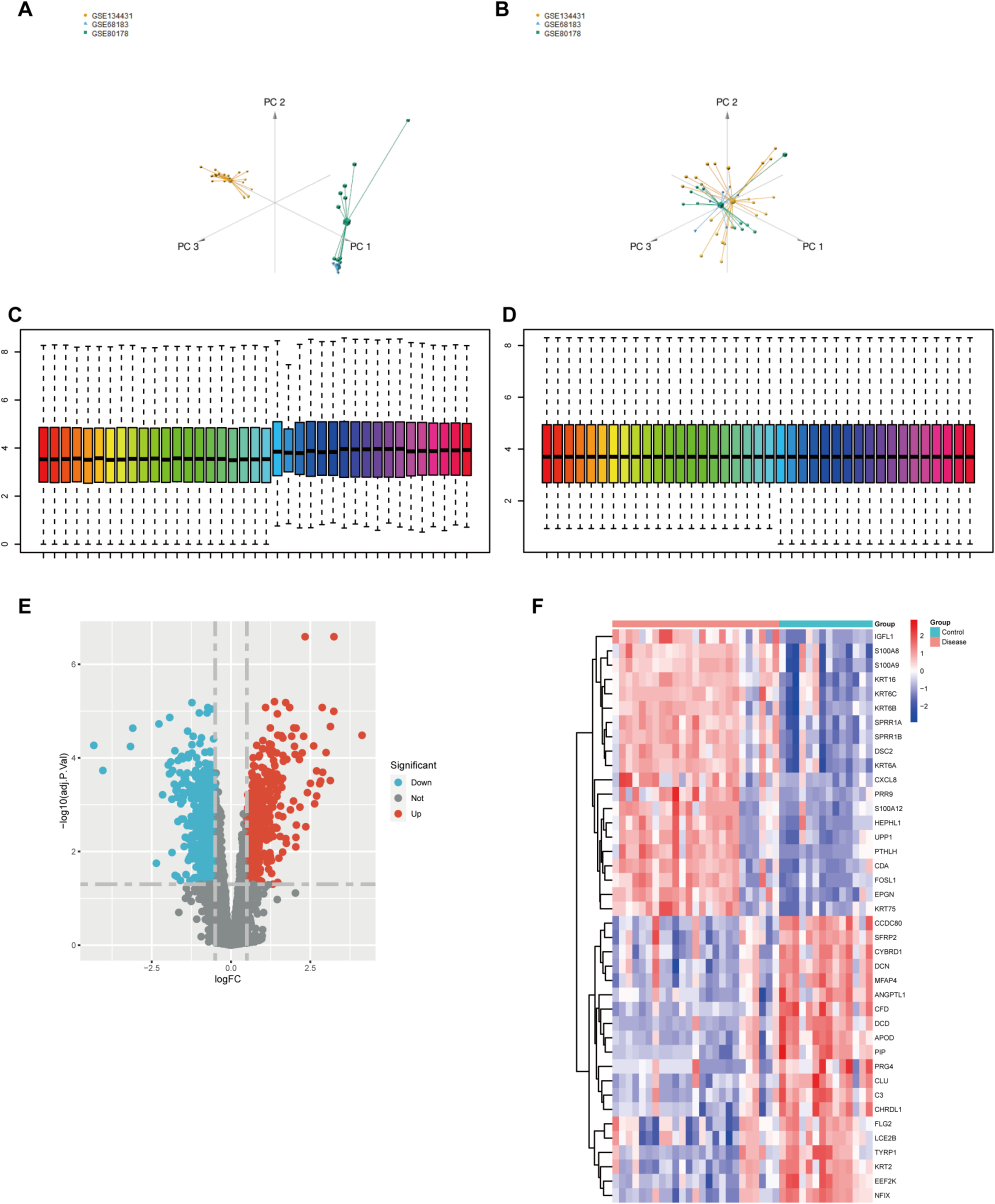
Normalization of GEO data and differential gene expression analysis. **(A)** PCA, Principal Component Analysis results before batch effect removal; **(B)** PCA results after batch effect removal; **(C)** Comparison of expression changes in a subset of gene sets reveals that batch effects still influence overall gene expression; **(D)** Normalization of the dataset results in more uniform overall gene expression. Differential expression analysis identifies upregulated and downregulated genes. **(E)** Volcano plot representing differential analysis results, with blue indicating downregulated genes and red indicating upregulated genes; **(F)** Genes showing differential expression between disease and healthy states, with red indicating higher expression and blue indicating lower expression, highlighting significant differences in gene expression between the two groups.

We integrated three DFU datasets from GEO: GSE68183, GSE80178, and GSE134431, resulting in a compiled dataset containing 15,151 genes and 39 samples. These samples included 14 normal skin tissues and 25 DFU tissues, originating from two distinct probe detection platforms. To address batch effects, we conducted principal component analysis (PCA), observing that samples were entirely separated along the PC1 axis in three-dimensional space based on the dataset source, indicating significant batch effects (**Figure 1A**). Post-removal of batch effects, the overall distribution of sample expression across the PCA space was homogenized (**Figure 1B**). We further verified the presence of batch effects by examining the expression variations in a subset of genes, finding that the overall gene expression still displayed batch-dependent influences (**Figure 1C**). Normalization of the dataset led to a more unified gene expression profile (**Figure 1D**).

Our differential expression analysis revealed a total of 1,234 differentially expressed genes (DEGs) in the combined dataset. Out of these, 560 genes were upregulated and 674 genes were downregulated in DFU samples compared to normal controls (**Figure 1E**). These identified DEGs allowed clear distinction between DFU samples and healthy tissues (**Figure 1F**).

### 3.2 Functional enrichment analysis of differentially expressed genes

**Figure 2:**
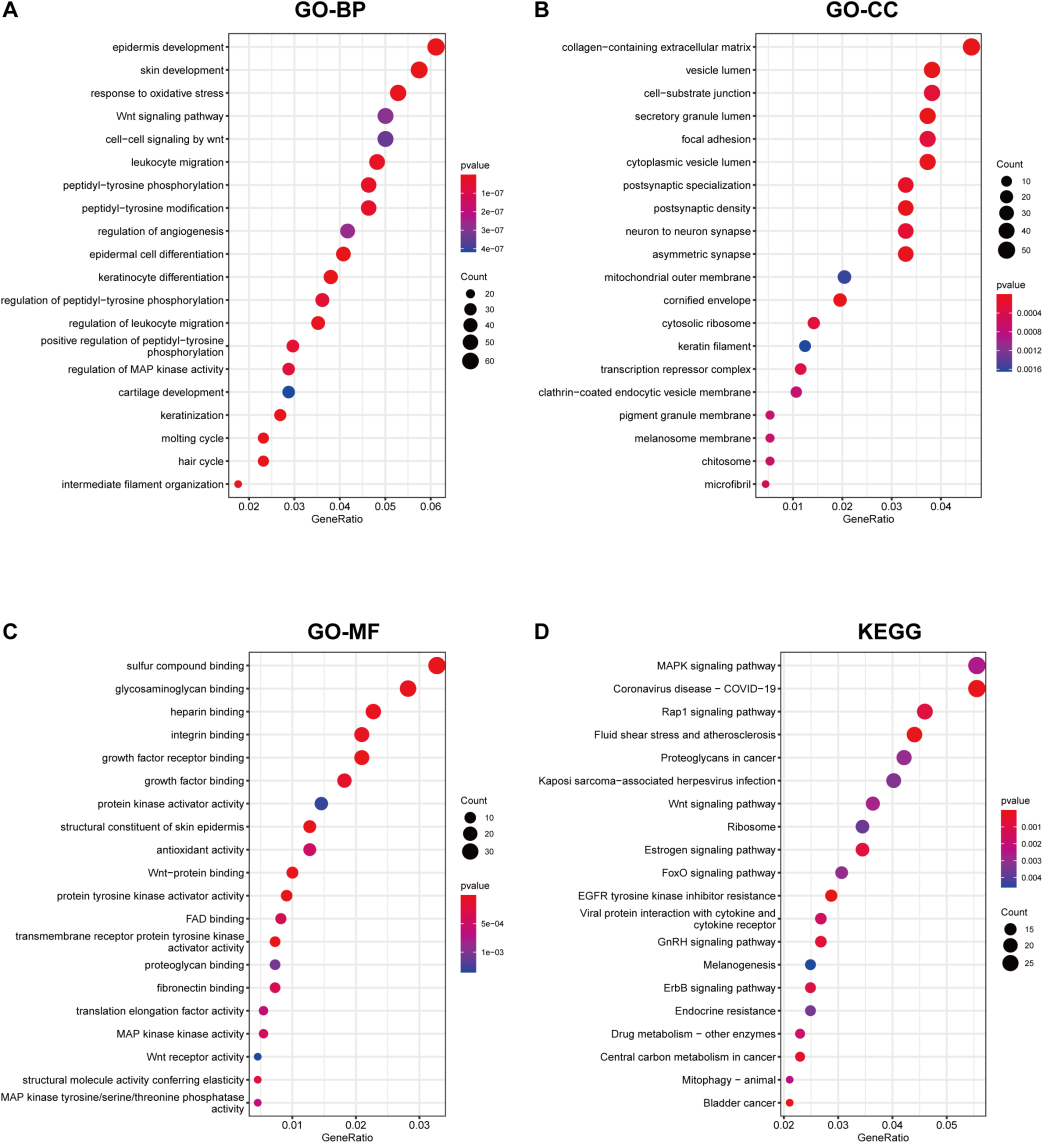
GO annotation and KEGG enrichment analysis of differential genes. GO annotation illustrates the functions of differentially expressed genes, including pathways annotated in BP, Biological Process **(A)**; CC, Cellular Component **(B)**; MF, Molecular Function **(C)**; and KEGG pathway enrichment analysis results **(D)**. These are ranked by GeneRatio within the pathway, with circle size representing the number of enriched genes and color indicating the p-value.

We performed functional annotation and enrichment analysis for the DEGs using Gene Ontology (GO) and KEGG pathway analysis. GO-Biological Process (BP) enrichment revealed that the DEGs were primarily involved in processes such as epidermis development (GO:0008544), skin development (GO:0043588), and response to oxidative stress (GO:0006979) (**Figure 2A**). GO-Cellular Component (CC) enrichment indicated enrichment in the collagen-containing extracellular matrix (GO:0062023), vesicle lumen (GO:0031983), and cell-substrate junction (GO:0030055) (**Figure 2B**). GO-Molecular Function (MF) analysis showed that these DEGs were enriched in functions such as sulfur compound binding (GO:1901681), glycosaminoglycan binding (GO:0005539), and heparin binding (GO:0008201) (**Figure 2C**). KEGG pathway enrichment analysis identified significant pathways, including the MAPK signaling pathway (hsa04010), Coronavirus disease - COVID-19 (hsa05171), and the Rap1 signaling pathway (hsa04015) (**Figure 2D**). These pathways illustrate the functional relevance of DEGs in the biological context of DFUs and controls.

### 3.3 Lactylation-related differentially expressed genes analysis

**Figure 3:**
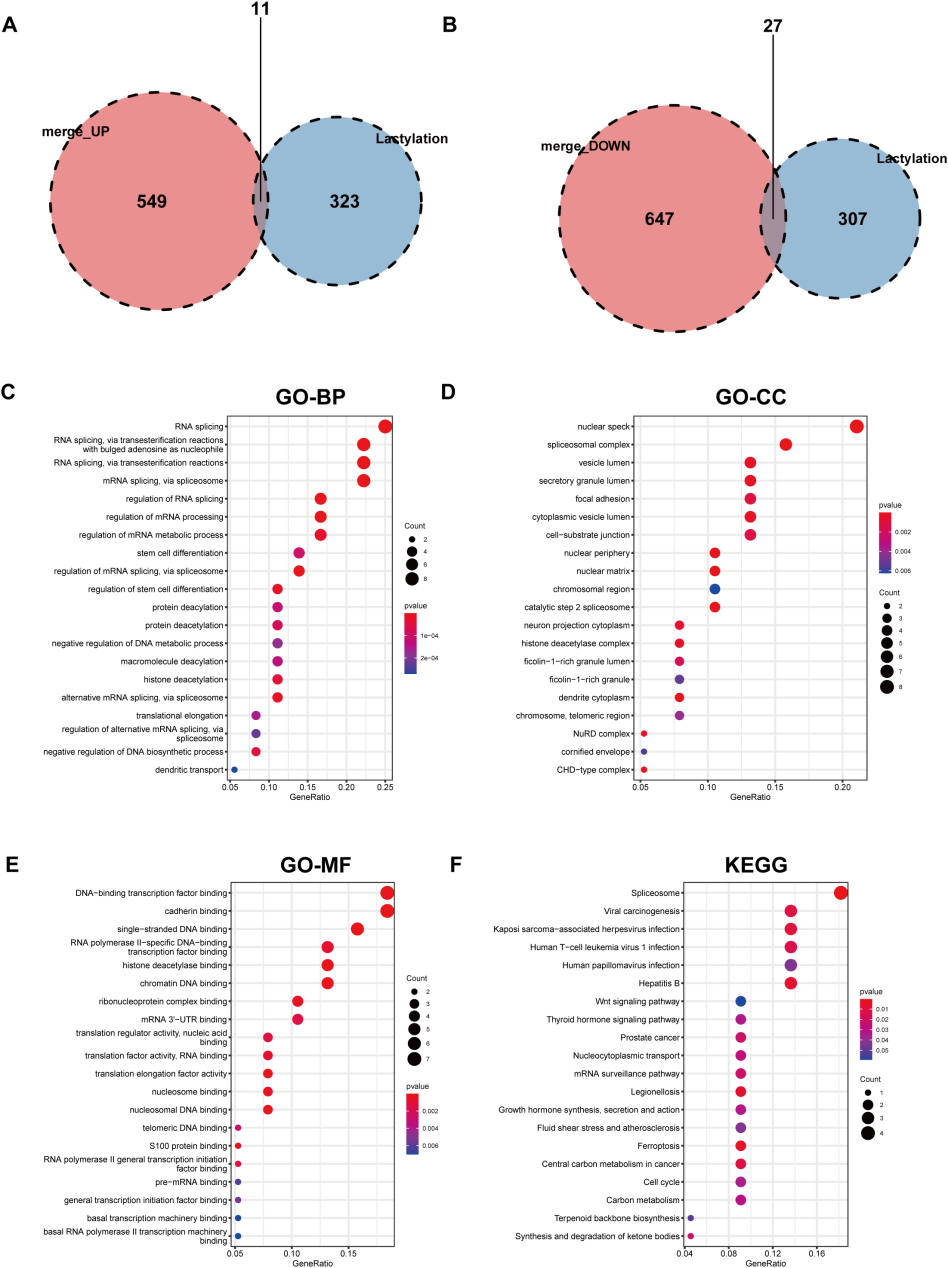
Analysis of lactylation-related differentially expressed genes. **(A)** Intersection of upregulated differential genes in DFU, identifying 11 differentially expressed genes; **(B)** Intersection of downregulated differential genes in DFU, identifying 27 differentially expressed genes; **(C)** Pathway annotation of these lactylation-related differential genes in BP, Biological Process; **(D)** Pathway annotation in CC, Cellular Component; **(E)** Pathway annotation in MF, Molecular Function; **(F)** Pathway annotation after KEGG enrichment of these lactylation-related differential genes.

We identified 336 lactylation-related genes from previous literature (PMID: 37242427, 35761067, 36092712) (**Supplementary Table S1**). Upon intersecting our DEGs with the lactylation gene set, we found 11 upregulated and 27 downregulated lactylation-related genes in DFUs (**Figures 3A, B**). Functional annotation of these lactylation-related DEGs revealed their involvement in biological processes such as RNA splicing (GO:0008380) (**Figure 3C**). Cellular component analysis indicated an enrichment in nuclear speck (GO:0016607) (**Figure 3D**). For molecular function, the genes were mostly engaged in DNA-binding transcription factor binding (GO:0140297) (**Figure 3E**). The KEGG pathway analysis highlighted the spliceosome pathway (hsa03040) (**Figure 3F**), indicating these lactylation-related DEGs are primarily associated with gene transcription and mRNA processing, ultimately affecting downstream gene expression and protein synthesis.

**Figure 4:**
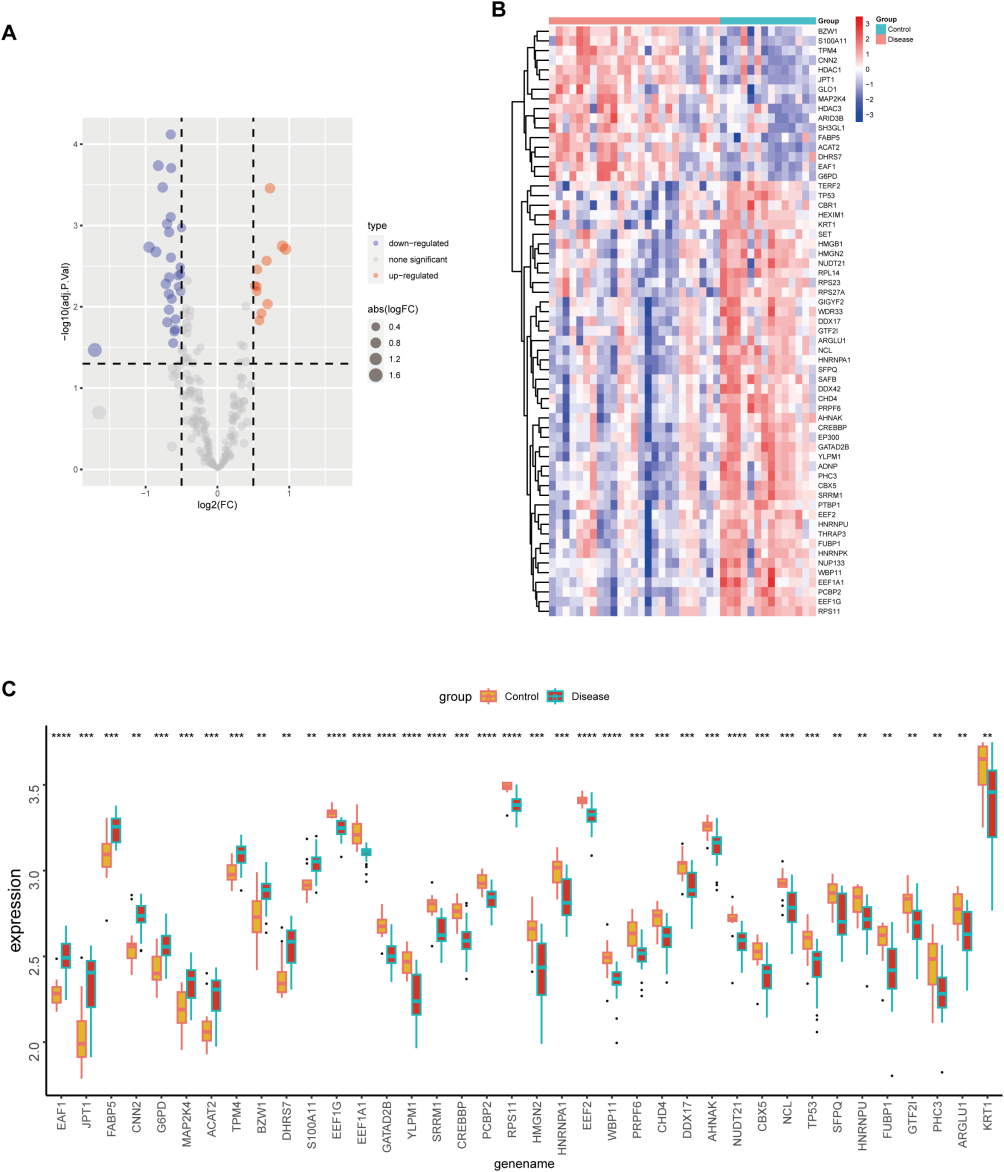
Expression differences of lactylation-related differentially expressed genes between disease and control groups. Volcano plot **(A)** and heatmap **(B)** display the expression differences of 38 intersected differentially expressed genes between disease and control groups; **(C)** Box plot showing expression levels and statistical differences of 38 differential genes between disease (green) and control groups (red). ns, p>0.05; *p<0.05, **p<0.01, ***p<0.001.

Analysis of the expression differences for the 38 intersected lactylation-related DEGs between DFUs and control groups revealed that most of these genes were significantly downregulated in DFUs (**Figures 4A, B**). Detailed expression profiles and statistical differences for these 38 DEGs are depicted (**Figure 4C**). The protein-protein interaction (PPI) network prediction for these genes demonstrated a substantial interaction network (**Supplementary Figure S1B**).

### 3.4 Identification of lactylation-related specific genes

**Figure 5:**
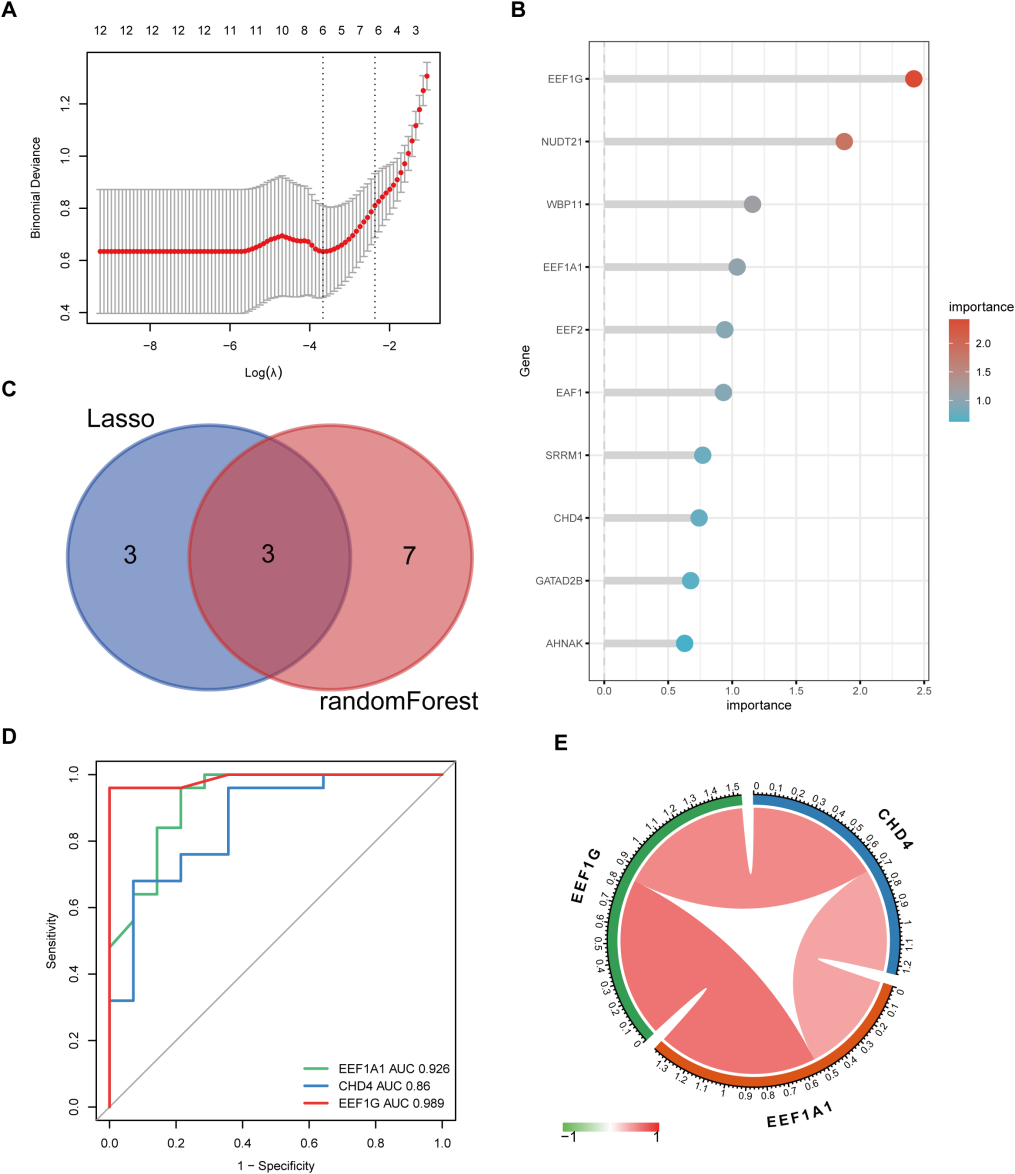
Machine learning identification of key lactylation-related genes in DFU. **(A)** LASSO regression machine learning identifies 6 key differential genes from 38 genes; **(B)** Random forest ranks and selects 10 genes based on importance; **(C)** Intersection of both methods identifies 3 core genes; **(D)** ROC curve and AUC indicate the discriminative ability of different genes for DFU; **(E)** Correlation among the 3 core genes (n=39, Spearman), with red indicating positive correlation and green indicating negative correlation.

To identify key differential genes in DFUs, we utilized machine learning algorithms comprising LASSO regression and random forest models. The LASSO regression identified six genes (MAP2K4, ACAT2, TPM4, EEF1G, EEF1A1, and CHD4) as significant markers (**Figure 5A**). Through the random forest algorithm, the following genes were selected as significant: EEF1G, NUDT21, WBP11, EEF1A1, EEF2, EAF1, SRRM1, CHD4, GATAD2B, and AHNAK (**Figure 5B**). Cross-referencing both methods, three core genes were identified: EEF1A1, CHD4, and EEF1G (**Figure 5C**). ROC curve analysis demonstrated the ability of these three genes to discriminate between DFU and control tissues with areas under the curve (AUC) of 0.926, 0.860, and 0.989, respectively (**Figure 5D**). We also investigated the correlation between the expressions of these three core genes and found that they were positively correlated (**Figure 5E**).

### 3.5 Immune function and immune cell infiltration analysis

**Figure 6:**
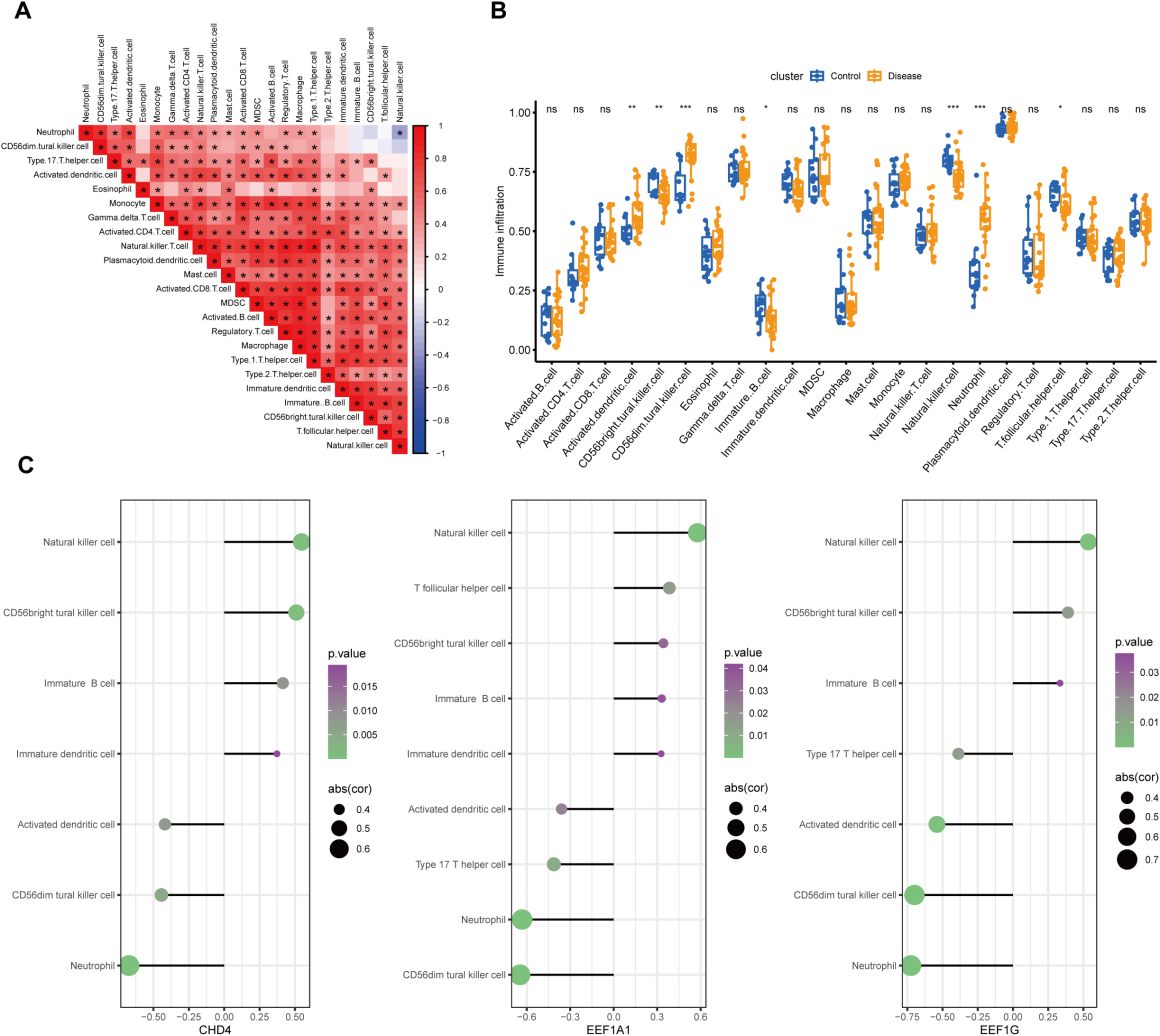
Immune function and immune cell infiltration analysis. **(A)** Correlation among different immune cell infiltrations; **(B)** Differences in immune cell infiltration between disease and control groups; **(C)** Correlation of 3 core genes with immune cell infiltration (only showing immune cells with p<0.05, plotted using ggplot2). Circle size indicates correlation coefficient, and color represents p-value. ns, p>0.05; *p<0.05, **p<0.01, ***p<0.001.

Previous studies have shown a close relationship between lactylation genes and inflammatory response and immune cell infiltration. Thus, we assessed the extent of immune cell infiltration in our study. Correlation analysis of immune cell infiltration in all samples indicated a generally positive correlation trend among different types of immune cells (**Figure 6A**). Comparing immune cell infiltration between DFU and control tissues revealed increased infiltration of Activated dendritic cells, CD56dim natural killer cells, and Neutrophils in DFUs. Conversely, there was a decreased infiltration of CD56bright natural killer cells, Immature B cells, Natural killer cells, and T follicular helper cells in DFUs (**Figure 6B**). Assessing the correlation between the core genes (EEF1A1, CHD4, and EEF1G) and immune cell infiltration suggested positive correlations with the infiltration of Natural killer cells and CD56bright natural killer cells, and negative correlations with the infiltration of Neutrophils and CD56dim natural killer cells (**Figure 6C**). To validate the robustness of our ssGSEA−based immune infiltration predictions, we performed cross−validation analyses including cell−type composition profiling, skin− and wound−specific gene signature sets, and the independent MCP−counter algorithm. These complementary approaches confirmed the directional consistency of the observed immune cell changes (e.g., reduced NK cell and increased neutrophil signals), as summarized in **Supplementary Figure S2**. These findings suggest that the three core genes significantly impact overall immune cell infiltration.

### 3.6 Analysis of disease-specific genes

**Figure 7:**
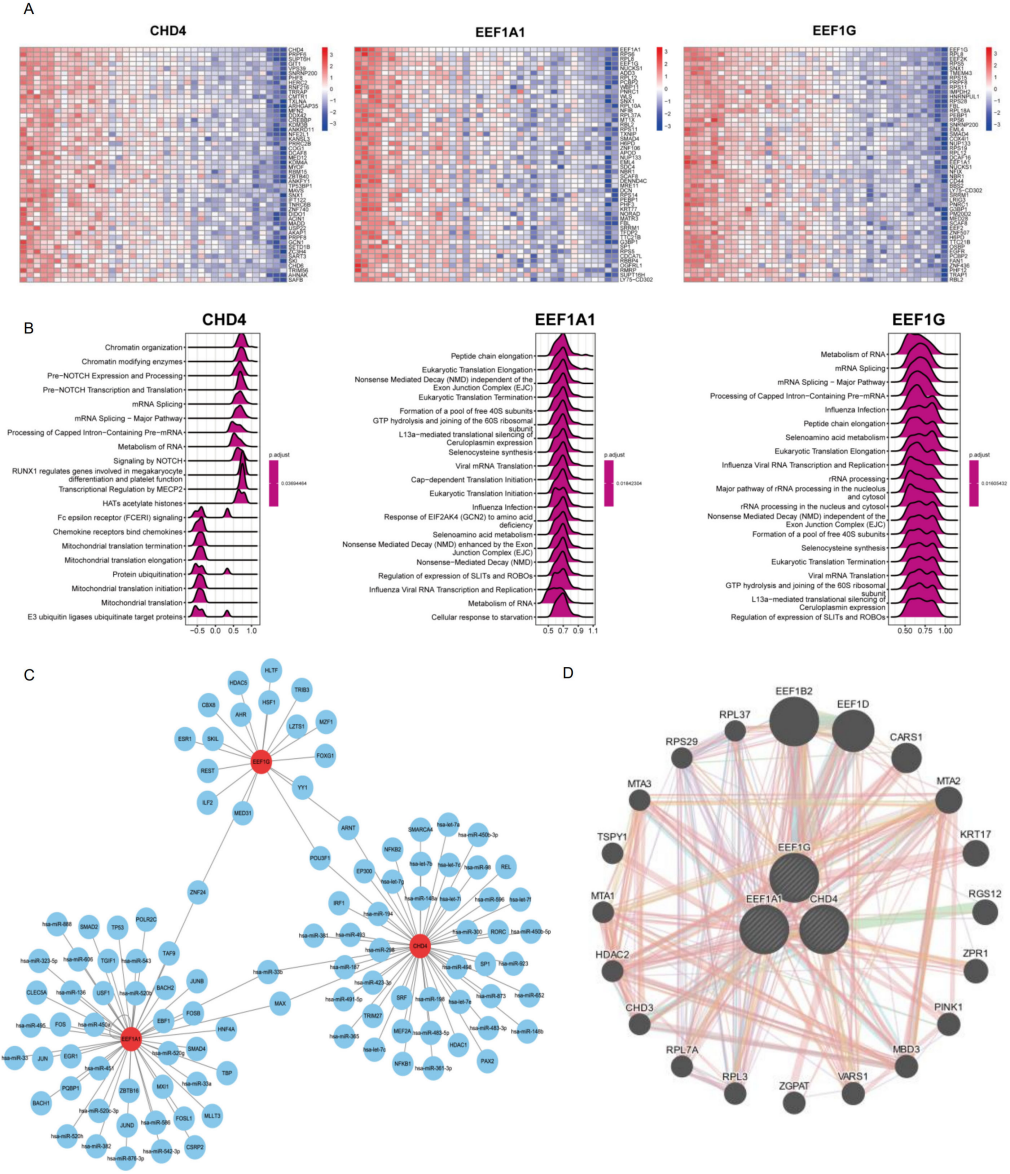
Integrative bioinformatic analysis of DFU−specific core genes (EEF1A1, CHD4, and EEF1G). **(A)** Correlation analysis of the three core genes with all other genes in the discovery cohort. Heatmap showing the top 50 genes positively correlated with each core gene (ranked by Spearman correlation coefficient). **(B)** GSEA, Gene set enrichment analysis based on the correlation results from panel **(A)**. The plot displays the top 20 Reactome pathways from single−gene GSEA for each core gene. ES, The enrichment scores are shown; positive scores indicate positive correlation with the pathway, and negative scores indicate negative correlation. **(C)** Prediction of upstream regulatory networks. TFs, Transcription factors and miRNAs, microRNAs targeting the three core genes were predicted using the RegNetwork database. Core genes are shown in red nodes. Only genes and miRNAs present in the database are displayed. **(D)** PPI, Protein−protein interaction network of the three core genes and their functionally interacting partners. The network was constructed using the GENEMANIA database, revealing shared functional pathways and co−expression relationships.

We further analyzed the correlation of the three core genes (EEF1A1, CHD4, and EEF1G) with all genes in different samples, displaying the expression of the top 50 positively correlated genes through heat maps (**Figure 7A**). GSEA analysis based on the correlation results of the three core genes indicated that CHD4 mainly promotes mRNA splicing and transcription initiation while inhibiting the degradation of translation-related proteins. EEF1A1 was found to mainly enhance protein translation initiation, and EEF1G was seen to promote both mRNA maturation and translation initiation (**Figure 7B**). These results are consistent with the known functions of these genes. Upstream miRNAs and transcription factors regulating the three core genes were predicted using the regnetwork database. Although there were some common regulatory factors, no single transcription factor was identified to regulate all three genes simultaneously (**Figure 7C**).

Analysis of the interactions between the three core genes and potential compounds revealed that 4-(5-benzo(1,3)dioxol-5-yl-4-pyridin-2-yl-1H-imidazol-2-yl)benzamide, (6-(4-(2-piperidin-1-ylethoxy)phenyl))-3-pyridin-4-ylpyrazolo(1,5-a)pyrimidine, vorinostat, Cadmium Chloride, and various Enzyme Inhibitors could closely interact with these target genes, suggesting their potential as gene function modulators (**Supplementary Figure S1C**). These in silico-predicted interactions provide testable hypotheses for future experimental validation. Using the GENEMANIA database to analyze the PPI interactions of these core genes, it became evident that they share numerous functional partners, implicating possible collaborative roles in gene regulation and function (**Figure 7D**).

### 3.7 *In vivo* experimental validation

To validate the expression changes of core lactylation-related genes identified through bioinformatic analysis under diabetic wound conditions, we conducted experimental validation using both *in vitro* cellular models and clinical tissue samples.

**Figure 8:**
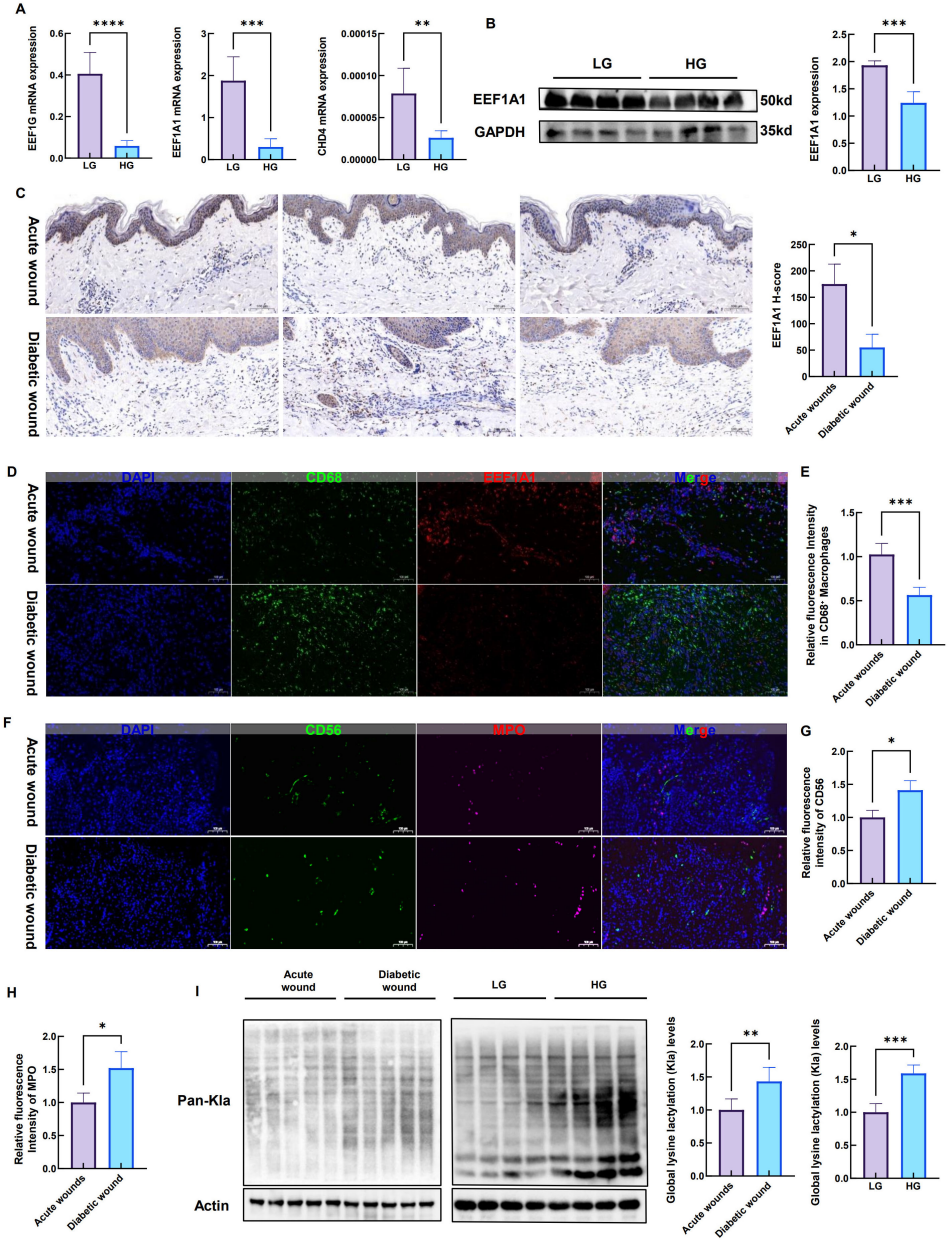
High glucose downregulates EEF1A1 expression in macrophages, alters immune cell infiltration, and elevates global lactylation in diabetic wound tissues. **(A)** qPCR analysis of *EEF1G*, *EEF1A1*, and *CHD4* mRNA expression levels in BMDMs, bone marrow-derived macrophages cultured under low glucose (LG, 5.5 mM) or high glucose (HG, 25 mM) conditions. All three genes were significantly downregulated in the HG group compared with the LG group (n = 5, **p < 0.01, ***p < 0.001, ****p < 0.0001). **(B)** Western blot analysis of EEF1A1 protein expression in BMDMs under LG or HG conditions. GAPDH served as the loading control. Left panel: representative immunoblot bands; right panel: densitometric quantification. High glucose significantly suppressed EEF1A1 protein expression (n = 4, ***p < 0.001). **(C)** Immunohistochemical staining of EEF1A1 in human acute wound tissues and DFU, diabetic foot ulcer tissues. EEF1A1-positive signals (brown) were markedly reduced in DFU tissues. H−score for EEF1A1 IHC was also statistically analyzed (n = 3, *p < 0.05) **(D)** Immunofluorescence co-localization of macrophages (CD68^+^, green) and EEF1A1 (red) in human wound tissues. Nuclei were counterstained with DAPI (blue). Merged images show yellow as co-localization. Representative images demonstrate substantially weaker EEF1A1 fluorescence intensity within CD68^+^ macrophages in DFU tissues compared with acute wounds. **(E)** Quantitative analysis of MFI, mean fluorescence intensity of EEF1A1 within CD68^+^ macrophages from the experiments shown in **(D)** (n = 3 per group, ***p < 0.001). **(F)** Immunofluorescence staining of NK, natural killer cells (CD56^+^, green) and neutrophils (MPO^+^, red) in human acute wound and DFU tissues. Nuclei were counterstained with DAPI (blue). Merged images show yellow as co-localization. **(G)** Quantitative analysis of CD56^+^ NK cell fluorescence intensity from the experiments shown in **(F)**. DFU tissues exhibited significantly reduced NK cell signal compared with acute wounds (n = 3 per group, *p < 0.05). **(H)** Quantitative analysis of MPO^+^ neutrophil fluorescence intensity from the experiments shown in **(F)**. DFU tissues showed significantly increased neutrophil signal compared with acute wounds (n = 3 per group, *p < 0.05). **(I)** Western blot analysis of global lysine lactylation (Kla) levels. Left panel: protein lysates from human acute wound and DFU tissues (n = 5 per group). Right panel: protein lysates from BMDMs cultured under LG or HG conditions (n = 4 per group). GAPDH served as the loading control. Significantly elevated Kla levels were observed in both DFU tissues (**p < 0.01) and HG treated BMDMs (***p < 0.001).

First, we stimulated mouse bone marrow-derived macrophages (BMDMs) with high glucose to mimic the inflammatory microenvironment of diabetic wounds. Quantitative real-time PCR (qRT-PCR) results revealed that, compared to normal glucose controls, high glucose treatment significantly downregulated mRNA expression levels of CHD4, EEF1A1, and EEF1G (**Figure 8A**), suggesting that hyperglycemic conditions may impair their biological functions by suppressing transcription.

Given the central role of EEF1A1 in protein translation and its highest classification performance in the discovery dataset among the identified genes in machine learning models (AUC = 0.989), we further validated its expression at the protein level. Western blot analysis demonstrated a significant reduction in EEF1A1 protein expression in BMDMs following high glucose exposure (**Figure 8B**), confirming impaired expression of this key factor under diabetic metabolic stress at the functional protein level.

To further assess the clinical relevance of these findings, we performed immunohistochemical analysis on human acute skin wound tissues and chronic diabetic foot ulcer (DFU) tissues. In acute wound tissues, EEF1A1 exhibited strong positive staining in newly formed epithelial cells and residual structures of skin appendages. In contrast, its expression was markedly reduced in the epidermal regions of diabetic chronic wounds (P<0.05) (**Figure 8C**), indicating that loss of EEF1A1 expression is closely associated with impaired re-epithelialization in diabetic ulcers.

Furthermore, we employed immunofluorescence double labeling to examine the specific expression of EEF1A1 within macrophage populations. Notably, CD68+ macrophage infiltration was significantly increased in diabetic wounds, reflecting sustained chronic inflammation. However, the fluorescent signal of EEF1A1 was substantially diminished, particularly within CD68+ macrophage-rich areas (**Figure 8D**). Analysis of mean fluorescence intensity (MFI) within CD68^+^ macrophages indicated a dramatically decreasing of EEF1A1 under DFU condition (P<0.001) (**Figure 8E**). These findings not only corroborate our *in vitro* results but also reveal that in the human DFU microenvironment, downregulation of EEF1A1 is closely linked to macrophage dysfunction and compromised wound repair.

Additionally, we performed immunofluorescence staining for CD56 (NK cells) and MPO (neutrophils) in human acute wound and DFU tissues (**Figure 8F**). Quantitative analysis revealed that CD56^+^ NK cell signals were significantly reduced in DFU tissues (P < 0.05), while MPO^+^ neutrophil signals were significantly increased (p < 0.05) (**Figures 8G, H**).We further examined global lysine lactylation (Kla) levels by Western blot. As shown in **Figure 8I**, DFU tissues exhibited significantly higher Kla levels than acute wound tissues (left panel, n=5, P < 0.01). Consistently, high−glucose−treated BMDMs showed elevated Kla levels compared with normal glucose controls (right panel, n=4, P < 0.001). These data confirm that lactylation is enhanced in the diabetic wound milieu and in hyperglycemia−stressed macrophages.

By conducting these analyses, this study provides an in-depth comparison between DFU and normal tissues at the transcriptomic level, highlighting the role of lactylation-related genes and their relationships with immune cell infiltration. Our findings underscore potential therapeutic targets that could be leveraged to develop new treatments for diabetic foot ulcers.

## 4 Discussion

Patients suffering from diabetic foot ulcers accompanied by lower extremity vascular disease often experience rapid progression and poor clinical outcomes, frequently leading to amputation if standardized treatment is not administered. Early diagnosis enables targeted interventions, which can prevent disease progression and reduce the risk of adverse consequences (26). Our study explored the profound role of lactylation-related genes in the pathogenesis of diabetic foot ulcers (DFUs). By analyzing transcriptomic data from GEO and identifying key regulatory targets and their interaction with immune cell infiltration, we have provided new insights into the molecular mechanisms underlying DFUs. This discussion delves deeper into the findings and implications of the study, specifically focusing on the crucial phases of wound healing, the role of lactylation, and the potential of identified core genes as therapeutic targets.

Wound healing is a complex process that typically follows a well-coordinated sequence of hemostasis, inflammation, proliferation, contraction, and remodeling (27). The hemostasis phase begins immediately after injury, where platelet aggregation and release of chemotactic factors initiate the clotting cascade to stop bleeding. Neutrophils then migrate to the wound site to clear debris and bacteria, followed by macrophage infiltration to engulf invading pathogens and damaged tissues. This inflammatory phase typically lasts around 72 hours (28). However, in diabetic wounds, there is impaired function and abnormal apoptosis of inflammatory cells, leading to chronic inflammation and a heightened risk of infection. Elevated levels of pro-inflammatory mediators further exacerbate the inflammation, hindering the transition to subsequent stages of wound healing (29).

During the proliferation phase, fibroblasts, keratinocytes, and endothelial cells proliferate, and extensive angiogenesis occurs. Proteoglycans, hyaluronic acid, collagen, and elastic fibers collectively form an extracellular matrix replacing the clot. Multiple cytokines and growth factors, including those from the TGF-β family, IL family, and angiogenesis factors, play pivotal roles in this phase (30). However, in the diabetic state, hyperglycemia, insulin resistance, and oxidative stress impede angiogenesis and cellular proliferation (31, 32). Consequently, the levels of several cytokines and growth factors are reduced, making effective wound healing challenging.

The final phase of wound healing is remodeling, wherein cell proliferation and apoptosis reach equilibrium. The blood supply to the damaged region stabilizes while the protein and collagen components of the extracellular matrix become more structured. This phase can last several months or even years. In diabetic wounds, however, persistent abnormal apoptosis and reduced collagen and protein synthesis prevent normal remodeling, leading to chronic wounds that are difficult to heal (33).

One of the most fascinating aspects of our research lies in the role of lactate and lactylation in wound healing. Lactate, the end-product of glycolysis, is integral to various stages of wound healing. Lactylation, as an epigenetic modifier, controls gene expression through histone lysine lactylation, leading to subsequent changes in post-translational modifications and downstream gene expression (6). These modifications trigger a cascade of biological events that promote or inhibit various cellular processes.

Emerging studies have found that lactylation levels correlate with the intensity of glycolysis within cells. For instance, hexokinase 2-mediated gene expression via histone lactylation is essential for hepatic stellate cell activation and liver fibrosis (34). This suggests that glycolysis can upregulate specific protein lactylations, affecting cell differentiation and immune responses. In CD4 T cells and TH17 cells, glycolysis-driven lactylation specifically modulates these cells’ differentiation, emphasizing the crucial role of this modification in immune cell function (35). Furthermore, lactylation 1inhibits the inflammatory metabolic adaptation in pro-inflammatory macrophages (36, 37). Given the critical roles of these cells in wound healing and the heightened inflammatory state in DFUs, understanding the underlying lactylation-driven mechanisms can offer new therapeutic insights. Lactate and lactylation, therefore, serve as pivotal modulators in the wound healing process by influencing immune regulation, inflammation control, and proliferation and remodeling stages.

Our study identified three core genes—EEF1A1, CHD4, and EEF1G—that play significant roles in DFU pathogenesis via lactylation modifications. Understanding their functions sheds light on how they might modulate key processes in DFU development and healing. CHD4, part of the CHD family, is a vital component of the nucleosome remodeling and deacetylase (NuRD) complex, a well-known transcriptional regulatory complex (38). As an ATP-dependent translocase, CHD4’s functionality has widespread implications in various biological contexts, including tumorigenesis (39). Prior studies have shown that proteins within the NuRD complex, including CHD4, undergo lactylation (40). This lactylation of CHD4 could regulate its activity and consequently affect gene expression and chromatin conformation. Given its central role in chromatin remodeling, lactylation of CHD4 might modulate transcriptional responses crucial for managing cellular stress and inflammatory responses in DFUs. In our study, CHD4 was one of the key differentially expressed genes in DFUs, thus supporting its potential role in regulating critical pathways involved in wound healing. Modifying CHD4 activity through lactylation could thereby influence gene transcription patterns necessary for effective healing in diabetic wounds.

EEF1A1 is one of the most abundant and evolutionarily conserved proteins in the eukaryotic proteome. It plays a pivotal role in translational elongation by promoting the binding of aminoacyl-tRNA to the ribosome (41). There are two isoforms in mammalian cells: EEF1A1 and EEF1A2, which share 98% similarity at the amino acid level but exhibit distinct expression patterns (42). Previous research indicates that overexpression of EEF1A1 can promote corticospinal axon repair post-injury. Furthermore, deacetylation of EEF1A1 facilitates transcriptional activation necessary for remyelination (43). EEF1A1’s involvement in DFUs is likely through its role in enhancing cellular protein synthesis, which is crucial for cell proliferation and tissue repair. Additionally, the lactylation of EEF1A1 might further modulate its activity, positively impacting the healing process in DFUs. Our study’s findings underscore EEF1A1 as a critical gene whose lactylation could support effective wound healing.

Similar to EEF1A1, EEF1G encodes a subunit of the elongation factor-1 complex responsible for the enzymatic transfer of aminoacyl-tRNA to the ribosome (44). The N-terminal glutathione S-transferase domain in EEF1G suggests its involvement in regulatory functions extending beyond protein synthesis (45). Downregulated expression of EEF1G in DFUs indicates a compromised capacity for adequate protein synthesis and regulation in these wounds. Furthermore, lactylation of EEF1G might modulate its regulatory functions, impacting downstream gene translation and protein synthesis critical for wound healing. The consistent expression patterns of EEF1G across different stages of wound repair further spotlight its role in orchestrating protein synthesis needed for effective tissue regeneration.

The identification of lactylation-related genes, particularly EEF1A1, CHD4, and EEF1G, in DFUs opens novel therapeutic possibilities. Understanding the exact mechanisms by which these genes influence wound healing through lactylation can inspire targeted therapeutic strategies. Potential interventions might involve manipulating the lactylation levels of these key proteins to enhance their beneficial roles in wound repair. For instance, small molecules or inhibitors that modulate the enzymes responsible for lactylating or delactylating these proteins could be developed (46).

Additionally, using the NetworkAnalyst database, we identified several compounds predicted to interact with the three core genes (e.g., vorinostat, cadmium chloride, and various enzyme inhibitors). The identified compounds such as vorinostat and specific enzyme inhibitors highlight the feasibility of targeting these pathways pharmacologically (47). By altering the lactylation status of EEF1A1, CHD4, and EEF1G, these compounds might modulate gene expression profiles, enhance protein synthesis, and promote effective immune cell function and tissue repair in DFUs. It is important to emphasize that these interactions are computationally predicted based on existing database entries and lack experimental validation; therefore, they should be regarded as hypothesis−generating observations that warrant further investigation in appropriate cellular or animal models.

Several limitations should be acknowledged. First, the sample size of the GEO discovery cohort (25 DFU vs. 14 normal) and our human tissue validation set (3 DFU vs. 3 control) is modest. Larger multicenter cohorts are needed to validate our findings. Second, immune infiltration was inferred from bulk transcriptomes using ssGSEA, which provides indirect estimates. Although we experimentally validated reduced CD56^+^ NK cells and increased MPO^+^ neutrophils by immunofluorescence (**Figures 8F–H**) and cross−validated with xCell, single−cell resolution studies are still required. Third, while we provide direct evidence of elevated global lactylation (Kla) in DFU tissues and high−glucose−treated macrophages (**Figure 8I**), we have not established causality. Importantly, due to resource constraints, we could not include human monocyte−derived macrophage data. Mouse BMDMs under high glucose do not fully recapitulate the human DFU microenvironment, and extrapolation to human pathophysiology requires caution. A further limitation is the absence of detailed clinical annotations (e.g., infection status, antibiotic treatment, wound age) in the public datasets, precluding adjustment for potential confounders. Future prospective cohorts with standardized clinical metadata are needed. Finally, the gene−compound network is purely in silico and awaits experimental validation. Despite these limitations, our study provides a hypothesis−generating framework for lactylation−associated genes in DFU.

Despite these promising findings, further validation is necessary. Large-scale studies involving diverse patient cohorts and experimental models are needed to confirm the roles of lactylation-related genes in DFU pathogenesis and healing. Investigating the dynamic interaction between lactylation and other post-translational modifications could offer a holistic view of the regulatory networks involved in DFUs. Moreover, exploring the precise mechanisms and conditions under which lactylation influences these core genes will be pivotal. Employing advanced techniques such as CRISPR-Cas9 for gene editing and RNA interference could provide insights into the functional consequences of modulating lactylation levels in these key genes (48).

## 5 Conclusion

Our study underscores the critical involvement of lactylation-related genes in DFU pathogenesis and their potential as therapeutic targets. By integrating transcriptomic data and employing robust analytical methodologies, we have identified key molecular players and pathways that could be harnessed for developing innovative treatments for DFUs. These findings pave the way for future research aimed at validating these targets and exploring therapeutic interventions that specifically modulate lactylation pathways. Enhanced understanding and effective treatments tailored toward these molecular targets could significantly improve clinical outcomes for patients suffering from diabetic foot ulcers.

## Data Availability

The original contributions presented in the study are included in the article/**Supplementary Material**. Further inquiries can be directed to the corresponding authors.

## Supplementary material

Supplementary Figure S1 Analytical workflow and analysis of protein-protein interaction and potential compound–gene interactions. **(A)** Schematic representation of data merging, batch correction, differential expression analysis, nested cross−validation (feature selection, model training, performance evaluation), and independent external validation. **(B)** The PPI, protein-protein interaction networks of the 38 genes of **Figure 4** obtained from the intersection were predicted using the STRING database (https://string-db.org/). **(C)** Analysis of potential compound–gene interactions. Gene−compound binding relationships were explored using the NetworkAnalyst database. Predicted compounds (e.g., vorinostat, cadmium chloride, enzyme inhibitors) with potential binding affinity to the core genes are shown.

Supplementary Figure S2 Cross-validation of immune infiltration analysis. **(A)** Cell-type composition heatmap based on skin/wound-specific marker genes (keratinocytes, fibroblasts, endothelial cells, ECM, and immune subsets). Structural signals dominate, providing tissue context. **(B)** MCP-counter analysis as an independent immune estimation method, confirming directional consistency with ssGSEA. **(C, D)** ssGSEA using a skin-tailored gene signature collection (skin-resident T cells, Langerhans cells, dermal DCs, M1/M2 macrophages, wound healing, angiogenesis, barrier function, NK cell and neutrophil signatures). Enrichment patterns were compared between DFU and control groups. **(E)** Spearman correlation between original immune ssGSEA scores and skin-tailored ssGSEA scores, showing consistent directional trends. **(F)** Spearman correlation between ssGSEA scores and MCP-counter, corroborating ssGSEA findings. Collectively, these cross-validation approaches support the trend-level reliability of the original ssGSEA-based findings.
