# Supplementary figures and images for: Expression of lactylation-related genes and their correlation with diabetic foot ulcer occurrence and immune infiltration
Source: Front Immunol. 2026 Apr 29;17:1765123. doi: 10.3389/fimmu.2026.1765123 (PMC13167497; doi:10.3389/fimmu.2026.1765123)

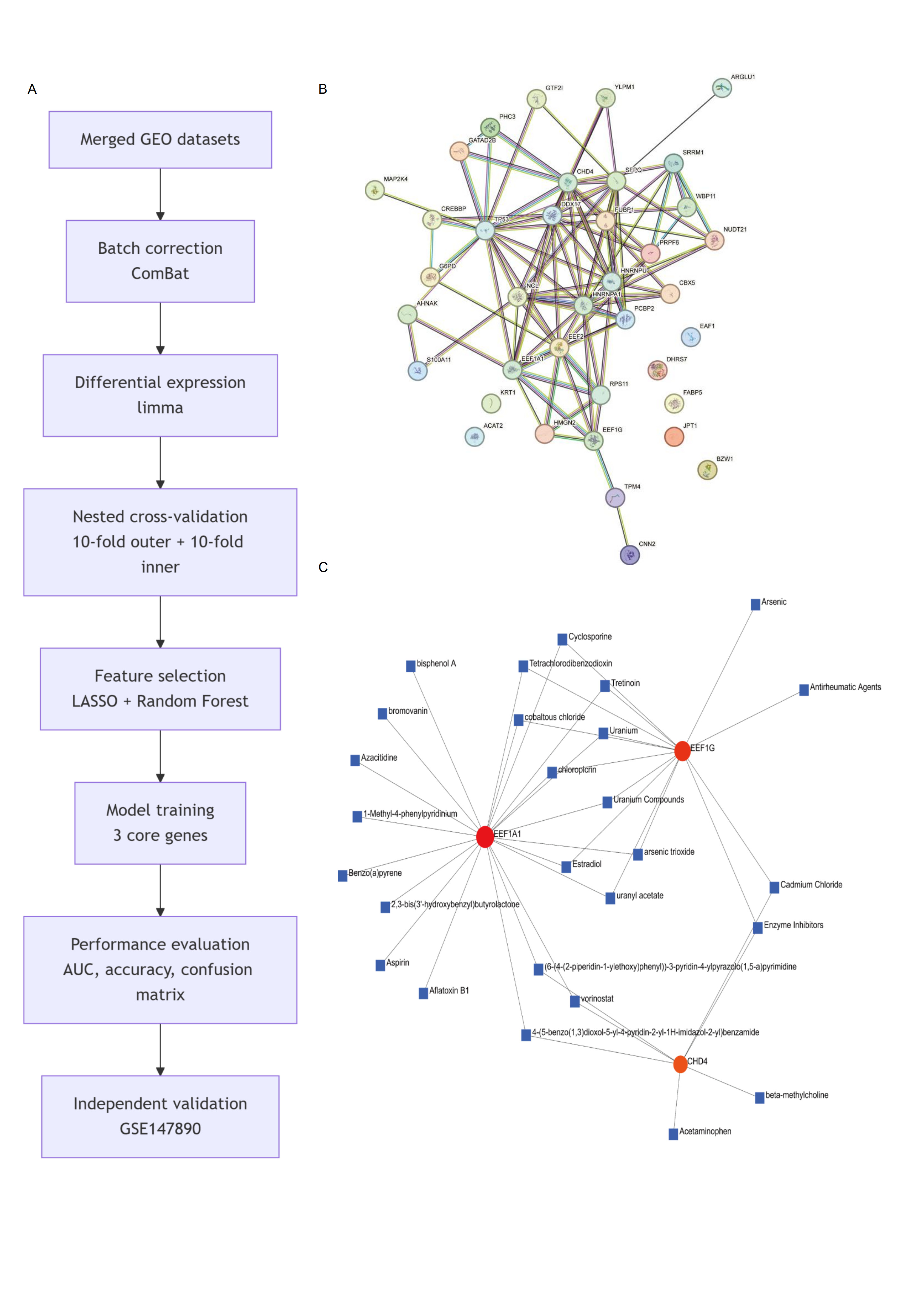

Supplement: Supplementary Figure S1 — Analytical workflow and analysis of protein-protein interaction and potential compound–gene interactions. (A) Schematic representation of data merging, batch correction, differential expression analysis, nested cross−validation (feature selection, model training, performance evaluation), and independent external validation. (B) The PPI, protein-protein interaction networks of the 38 genes of Figure 4 obtained from the intersection were predicted using the STRING database (https://string-db.org/). (C) Analysis of potential compound–gene interactions. Gene−compound binding relationships were explored using the NetworkAnalyst database. Predicted compounds (e.g., vorinostat, cadmium chloride, enzyme inhibitors) with potential binding affinity to the core genes are shown. [file Image1.jpeg]

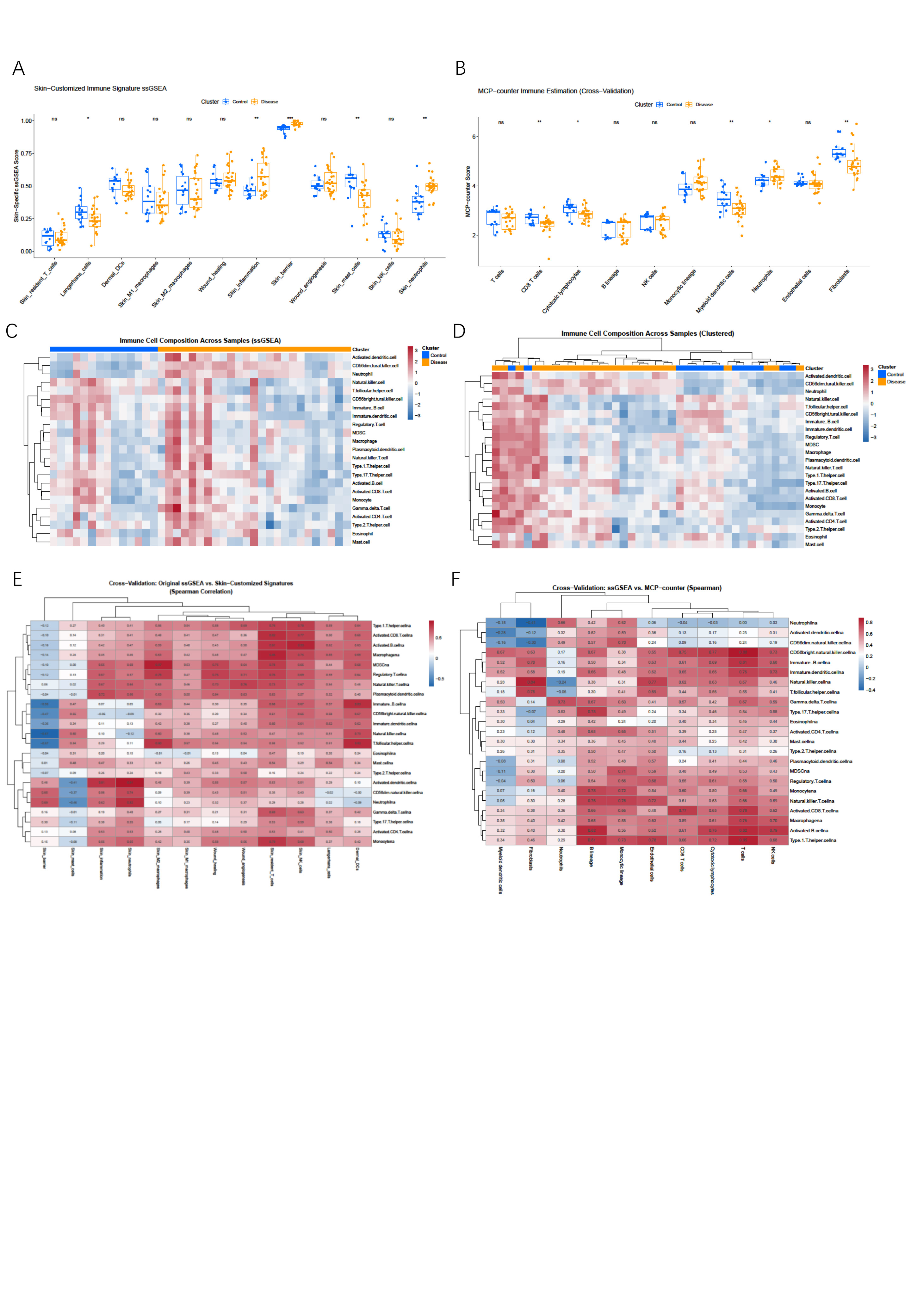

Supplement: Supplementary Figure S2 — Cross-validation of immune infiltration analysis. (A) Cell-type composition heatmap based on skin/wound-specific marker genes (keratinocytes, fibroblasts, endothelial cells, ECM, and immune subsets). Structural signals dominate, providing tissue context. (B) MCP-counter analysis as an independent immune estimation method, confirming directional consistency with ssGSEA. (C, D) ssGSEA using a skin-tailored gene signature collection (skin-resident T cells, Langerhans cells, dermal DCs, M1/M2 macrophages, wound healing, angiogenesis, barrier function, NK cell and neutrophil signatures). Enrichment patterns were compared between DFU and control groups. (E) Spearman correlation between original immune ssGSEA scores and skin-tailored ssGSEA scores, showing consistent directional trends. (F) Spearman correlation between ssGSEA scores and MCP-counter, corroborating ssGSEA findings. Collectively, these cross-validation approaches support the trend-level reliability of the original ssGSEA-based findings. [file Image2.jpeg]
